# MetaVal: Witness Validation via Verification

**DOI:** 10.1007/978-3-030-53291-8_10

**Published:** 2020-06-16

**Authors:** Dirk Beyer, Martin Spiessl

**Affiliations:** 8 Microsoft Research Lab, Redmond, WA USA; 9 University of Southern California, Los Angeles, CA USA; LMU Munich, Munich, Germany

**Keywords:** Computer-aided verification, Software verification, Program analysis, Software model checking, Certification, Verification witnesses, Validation of verification results, Reducer

## Abstract

Witness validation is an important technique to increase trust in verification results, by making descriptions of error paths (violation witnesses) and important parts of the correctness proof (correctness witnesses) available in an exchangeable format. This way, the verification result can be validated independently from the verification in a second step. The problem is that there are unfortunately not many tools available for witness-based validation of verification results. We contribute to closing this gap with the approach of *validation via verification*, which is a way to automatically construct a set of validators from a set of existing verification engines. The idea is to take as input a specification, a program, and a verification witness, and produce a new specification and a transformed version of the original program such that the transformed program satisfies the new specification if the witness is useful to confirm the result of the verification. Then, an ‘off-the-shelf’ verifier can be used to validate the previously computed result (as witnessed by the verification witness) via an ordinary verification task. We have implemented our approach in the validator  , and it was successfully used in SV-COMP 2020 and confirmed 3 653 violation witnesses and 16 376 correctness witnesses. The results show that  improves the effectiveness (167 uniquely confirmed violation witnesses and 833 uniquely confirmed correctness witnesses) of the overall validation process, on a large benchmark set. All components and experimental data are publicly available.

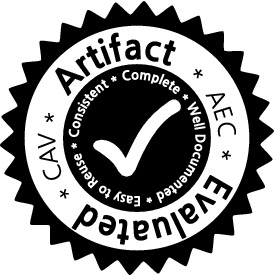



## Introduction

Formal software verification becomes more and more important in the development process for software systems of all types. There are many verification tools available to perform verification [4]. One of the open problems that was addressed only recently is the topic of results validation [10–12, 37]: The verification work is often done by untrusted verification engines, on untrusted computing infrastructure, or even on approximating computation systems, and static-analysis tools suffer from false positives that engineers in practice hate because they are tedious to refute [20]. Therefore, it is necessary to validate verification results, ideally by an independent verification engine that likely does not have the same weaknesses as the original verifier. Witnesses also help serving as an interface to the verification engine, in order to overcome integration problems [1].

The idea to witness the correctness of a program by annotating it with assertions is as old as programming [38], and from the beginning of model checking it was felt necessary to witness counterexamples [21]. Certifying algorithms [30] are not only computing a solution but also produce a witness that can be used by a computationally much less expensive checker to (re-)establish the correctness of the solution. In software verification, witnesses became standardized^1^ and exchangeable about five years ago [10, 11]. In the meanwhile, the exchangeable witnesses can be used also for deriving tests from witnesses [12], such that an engineer can study an error report additionally with a debugger. The ultimate goal of this direction of research is to obtain witnesses that are certificates and can be checked by a fully trusted validator based on trusted theorem provers, such as Coq and Isabelle, as done already for computational models that are ‘easier’ than C programs [40].

Yet, although considered very useful, there are not many witness validators available. For example, the most recent competition on software verification (SV-COMP 2020)^2^ showcases 28 software verifiers but only 6 witness validators. Two were published in 2015 [11], two more in 2018 [12], the fifth in 2020 [37], and the sixth is  , which we describe here. Witness validation is an interesting problem to work on, and there is a large, yet unexplored field of opportunities. It involves many different techniques from program analysis and model checking. However, it seems that this also requires a lot of engineering effort.

**Fig. 1.**
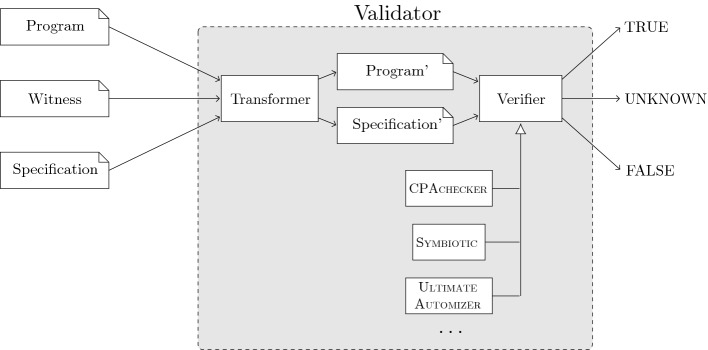
Validator construction using readily available verifiers

Our solution *validation via verification* is a construction that takes as input an off-the-shelf software verifier and a new program transformer, and composes a witness validator in the following way (see Fig. 1): First, the transformer takes the original input program and transforms it into a new program. In case of a violation witness, which describes a path through the program to a specific program location, we transform the program such that all parts that are marked as unnecessary for the path by the witness are pruned. This is similar to the reducer for a condition in reducer-based conditional model checking [14]. In case of a correctness witness, which describes invariants that can be used in a correctness proof, we transform the program such that the invariants are asserted (to check that they really hold) and assumed (to use them in a re-constructed correctness proof). A standard verification engine is then asked to verify that (1) the transformed program contains a feasible path that violates the original specification (violation witness) or (2) the transformed program satisfies the original specification and all assertions added to the program hold (correctness witness).

 is an implementation of this concept. It performs the transformation according to the witness type and specification, and can be configured to use any of the available software verifiers^3^ as verification backend.

**Contributions.**  contributes several important benefits:

- The program transformer was a one-time effort and is available from now on.
- Any existing standard verifier can be used as verification backend.
- Once a new verification technology becomes available in a verification tool, it can immediately be turned into a validator using our new construction.
- Technology bias can be avoided by complementing the verifier by a validator that is based on a different technology.
- Selecting the strongest verifiers (e.g., by looking at competition results) can lead to strong validators.
- All data and software that we describe are publicly available (see Sect. 6).

## Preliminaries

For the theoretical part, we will have to set a common ground for the concepts of verification witnesses [10, 11] as well as reducers [14]. In both cases, programs are represented as control-flow automata (CFAs). A *control-flow automaton* $C=(L,l_0,G)$ consists of a set *L* of control locations, an initial location ${l_0 \in L}$, and a set $G\subseteq L \times Ops \times L$ of control-flow edges that are labeled with the operations in the program. In the mentioned literature on witnesses and reducers, a simple programming language is used in which operations are either assignments or assumptions over integer variables. Operations $op \in Ops$ in such a language can be represented by formulas in first order logic over the sets *V*,$V'$ of program variables before and after the transition, which we denote by $op(V,V')$. In order to simplify our construction later on, we will also allow mixed operations of the form $f(V) \wedge \left( x' = g(V)\right)$ that combine assumptions with an assignment, which would otherwise be represented as an assumption followed by an assignment operation.

The conversion from the source code into a CFA and vice versa is straight forward, provided that the CFA is deterministic. A CFA is called *deterministic* if in case there are multiple outgoing CFA edges from a location *l*, the assumptions in those edges are mutually exclusive (but not necessarily exhaustive).

**Fig. 2.**
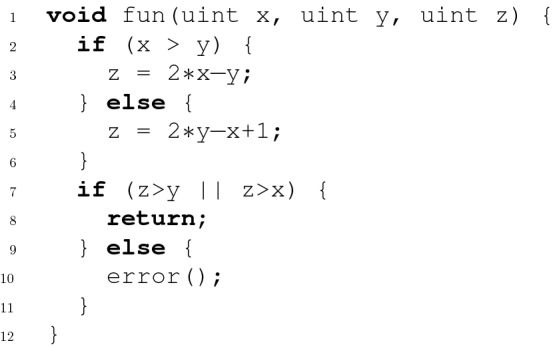
Example program for both correctness and violation witness validation

**Fig. 3.**
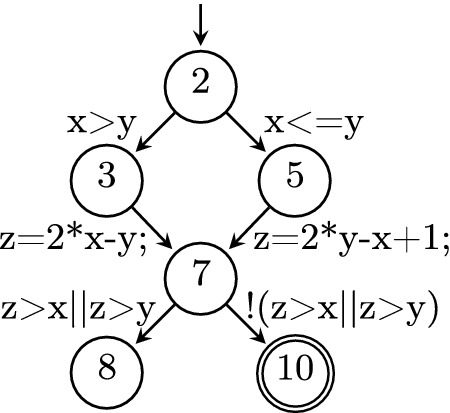
CFA *C* of example program from Fig. 2

Since our goal is to validate (i.e., prove or falsify) the statement that a program fulfills a certain specification, we need to additionally model the property to be verified. For properties that can be translated into non-reachability, this can be done by defining a set $T \subseteq L$ of target locations that shall not be reached. For the example program in Fig. 2 we want to verify that the call in line 10 is not reachable. In the corresponding CFA in Fig. 3 this is represented by the reachability of the location labeled with 10. Depending on whether or not a verifier accounts for the overflow in this example program, it will either consider the program safe or unsafe, which makes it a perfect example that can be used to illustrate both correctness and violation witnesses.

In order to reason about the soundness of our approach, we need to also formalize the program semantics. This is done using the concept of concrete data states. A *concrete data state* is a mapping from the set *V* of program variables to their domain $\mathbb {Z}$, and a *concrete state* is a pair of control location and concrete data state. A *concrete program path* is then defined as a sequence $\pi = (c_0,l_0)\xrightarrow {g_1}\dots \xrightarrow {g_n}(c_n,l_n)$ where $c_0$ is the initial concrete data state, $g_i = (l_{i-1}, op_i, l_i) \in G$, and $c_{i-1}(V),c_i(V') \vDash op_i$. A *concrete execution* $ex(\pi )$ is then derived from a path $\pi$ by only looking at the sequence $(c_0,l_0)\dots (c_n,l_n)$ of concrete states from the path. Note the we deviate here from the definition given in [14], where concrete executions do not contain information about the program locations. This is necessary here since we want to reason about the concrete executions that fulfill a given non-reachability specification, i.e., that never reach certain locations in the original program.

Witnesses are formalized using the concept of protocol automata [11]. A *protocol automaton* $W=(Q,\varSigma ,\delta ,q_0,F)$ consists of a set *Q* of states, a set of transition labels $\varSigma = 2^G \times \varPhi$, a transition relation $\delta \subseteq Q \times \varSigma \times Q$, an initial state $q_0$, and a set $F \subseteq Q$ of final states. A state is a pair that consists of a name to identify the state and a predicate over the program variables *V* to represent the state invariant.^4^ A transition label is a pair that consists of a subset of control-flow edges and a predicate over the program variables *V* to represent the guard condition for the transition to be taken. An *observer automaton* [11, 13, 32, 34, 36] is a protocol automaton that does not restrict the state space, i.e., if for each state $q \in Q$ the disjunction of the guard conditions of all outgoing transitions is a tautology. Violation witnesses are represented by protocol automata in which all state invariants are *true*. Correctness witnesses are represented by observer automata in which the set of final states is empty.

## Approach

### From Witnesses to Programs

When given a CFA *C* = $(L,l_0,G)$, a specification *T* $\subseteq$ *L*, and a witness automaton *W* = $(Q,\varSigma ,\delta ,q_0,F)$, we can construct a product automaton ${A_{C\times W} =(L\times Q,(l_0,q_0),\varGamma ,T \times F)}$ where $\varGamma \subseteq (L\times Q)\times (Ops\times \varPhi ) \times (L \times Q)$. The new transition relation $\varGamma$ is defined by allowing for each transition *g* in the CFA only those transitions $(S,\varphi )$ from the witness where $g \in S$ holds:

$$\begin{aligned} \varGamma = \left\{ \big ((l_i,q_i),(op,\varphi ),(l_j,q_j)\big )~\big \vert ~ \exists S:\big (q_i,(S,\varphi ),q_j\big ) \in \delta , (l_i,op,l_j) \in S\right\} \end{aligned}$$

We can now define the semantics of a witness by looking at the paths in the product automaton and mapping them to concrete executions in the original program. A path of the product automaton $A_{C,W}$ is a sequence ${(l_0,q_0)\xrightarrow {\alpha _0}\dots \xrightarrow {\alpha _{n-1}} (l_n,q_n)}$ such that ${\big ((l_i,q_i),\alpha _i,(l_{i+1},q_{i+1})\big ) \in \varGamma }$ and ${\alpha _i=(op_i,\phi _i)}$.

It is evident that the automaton $A_{C \times W}$ can easily be mapped to a new program $C_{C\times W}$ by reducing the pair $(op,\varphi )$ in its transition relation to an operation $\overline{op}$. In case *op* is a pure assumption of the form *f*(*V*) then $\overline{op}$ will simply be $f(V) \wedge \varphi (V)$. If *op* is an assignment of the form $f(V) \wedge \left( x' = g(V)\right)$, then $\overline{op}$ will be $(f(V)\wedge \varphi (V)) \wedge \left( x' = g(V)\right)$. This construction has the drawback that the resulting CFA might be non-deterministic, but this is actually not a problem when the corresponding program is only used for verification. The non-determinism can be expressed in the source code by using non-deterministic values, which are already formalized by the community and established in the SV-COMP rules, and therefore also supported by all participating verifiers. The concrete executions of $C_{C\times W}$ can be identified with concrete executions of *C* by projecting their pairs (*l*, *q*) on their first element. Let $proj_C(ex(C_{C\times W}))$ denote the set of concrete executions that is derived this way. Due to how the relation $\varGamma$ of $A_{C \times W}$ is constructed, it is guaranteed that this is a subset of the executions of *C*, i.e., $proj_C(ex(C_{C\times W})) \subseteq ex(C)$. In this respect the witness acts in very much the same way as a reducer [14], and the reduction of the search space is also one of the desired properties of a validator for violation witnesses.

### Programs from Violation Witnesses

**Fig. 4.**
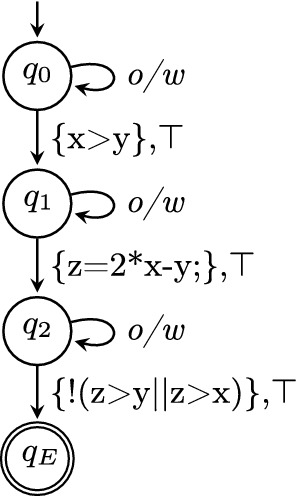
Violation witness $$W_V$$

**Fig. 5.**
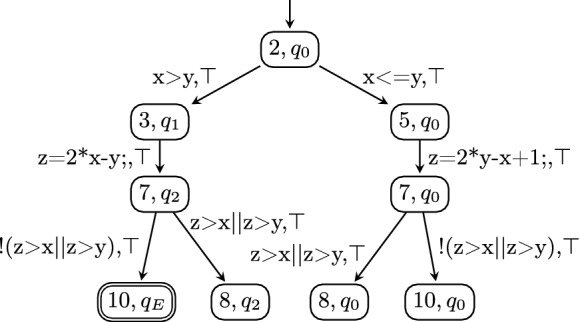
Product automaton $$A_{C \times W_V}$$

For explaining the validation of results based on a violation witness, we consider the witness in Fig. 4 for our example C program in Fig. 2. The program $C_{C\times W_V}$ resulting from product automaton $A_{C\times W_V}$ in Fig. 5 can be passed to a verifier. If this verification finds an execution that reaches a specification violation, then this violation is guaranteed to be also present in the original program. There is however one caveat: In the example in Fig. 5, a reachable state $(10,q_0)$ at program location 10 (i.e., a state that violates the specification) can be found that is not marked as accepting state in the witness automaton $W_V$. For a strict version of witness validation, we can remove all states that are in $T\times Q$ but not in $T \times F$ from the product automaton, and thus, from the generated program. This will ensure that if the verifier finds a violation in the generated program, the witness automaton also accepts the found error path. The version of  that was used in SV-COMP 2020 did not yet support strict witness validation.

### Programs from Correctness Witnesses

**Fig. 6.**
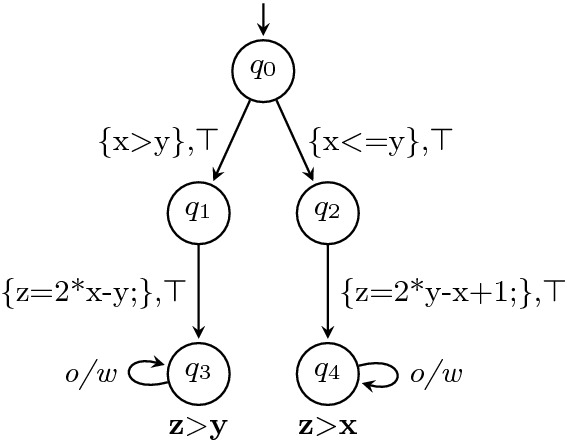
Correctness witness $$W_C$$

**Fig. 7.**
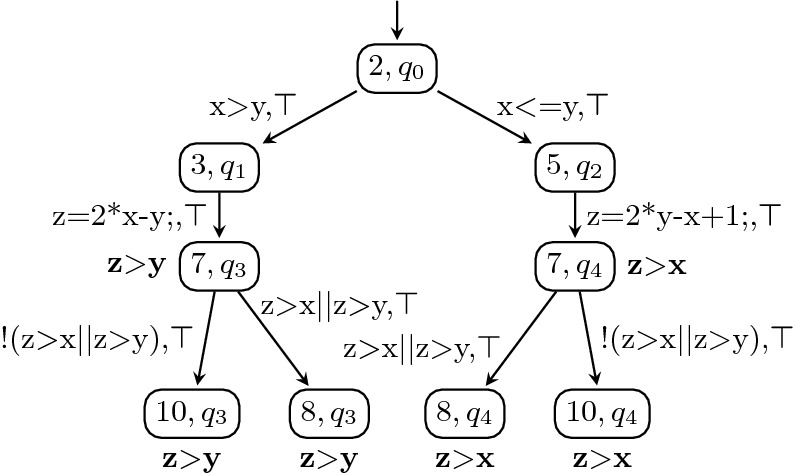
Product automaton $$A_{C \times W_C}$$

Correctness witnesses are represented by observer automata. Figure 6 shows a potential correctness witness $W_C$ for our example program *C* in Fig. 2, where the invariants are annotated in bold font next to the corresponding state. The construction of the product automaton $A_{C\times W_C}$ in Fig. 7 is a first step towards reestablishing the proof of correctness: the product states tell us to which control locations of the CFA for the program the invariants from the witness belong.

The idea of a result validator for correctness witnesses is to

1. check the invariants in the witness and
2. use the invariants to establish that the original specification holds.

We can achieve the second goal by extracting the invariants from each state in the product automaton $A_{C\times W_C}$ and adding them as conditions to all edges by which the state can be reached. This will then be semantically equivalent to assuming that the invariants hold at the state and potentially make the consecutive proof easier. For soundness we need to also ensure the first goal. To achieve that, we add transitions into a (new) accepting state from $T\times F$ whenever we transition into a state *q* and the invariant of *q* does not hold, and we add self-loops such that the automaton stays in the new accepting state forever. In sum, for each invariant, there are two transitions, one with the invariant as guard (to assume that the invariant holds) and one with the negation of the invariant as guard (to assert that the invariant holds, going to an accepting (error) state if it does not hold). This transformation ensures that the resulting automaton after the transformation is still a proper observer automaton.

## Evaluation

This section describes the results that were obtained in the 9th Competition on Software Verification (SV-COMP 2020), in which  participated as validator. We did not perform a separate evaluation because the results of SV-COMP are complete, accurate, and reproducible; all data and tools are publicly available for inspection and replication studies (see data availability in Sect. 6).

### Experimental Setup

**Execution Environment.** In SV-COMP 2020, the validators were executed in a benchmark environment that makes use of a cluster with 168 machines, each of them having an Intel Xeon E3-1230 v5 CPU with 8 processing units, 33 GB of RAM, and the GNU/Linux operating system Ubuntu 18.04. Each validation run was limited to 2 processing units and 7 GB of RAM, in order to allow up to 4 validation runs to be executed on the same machine at the same time. The time limit for a validation run was set to 15 min for correctness witnesses and to 90 s for violation witnesses. The benchmarking framework  2.5.1 was used to ensure that the different runs do not influence each other and that the resource limits are measured and enforced reliably [15]. The exact information to replicate the runs of SV-COMP 2020 can be found in Sect. 3 of the competition report [4].

**Benchmark Tasks.** The verification tasks^5^ of SV-COMP can be partitioned wrt. their specification into ReachSafety, MemSafety, NoOverflows, and Termination. Validators can be configured using different options for each specification.

**Table 1.** Overview of validation for violation witnesses in SV-COMP 2020

Specification	Measure		CPA-wtt	FShell-wtt			UAutomizer
ReachSafety (35 652 witnesses)	executed on	35 652	25 812	25 812	35 652	21 636	25 812
	uniquely confirmed	3 043	42	175	44	398	547
	jointly confirmed	8 019	6 010	6 740	1 566	8 055	3 802
Termination (9 720 witnesses)	executed on	3 043			9 720		9 720
	uniquely confirmed	566			9		235
	jointly confirmed	1 539			256		1 493
NoOverflow (3 149 witnesses)	executed on	3 149	3 149	3 149	3 149		3 149
	uniquely confirmed	6	1	31	1		89
	jointly confirmed	1 668	1 067	1 267	1 186		1 590
MemSafety (2 681 witnesses)	executed on	2 681	2 213	2 681	2 681		2 681
	uniquely confirmed	278	0	21	113		44
	jointly confirmed	737	250	364	478		372

**Table 2.** Overview of validation for correctness witnesses in SV-COMP 2020

Specification	Measure			UAutomizer
ReachSafety (66 435 witnesses)	executed on	66 435	66 435	66 435
	uniquely confirmed	1 750	391	708
	jointly confirmed	17 592	13 862	16 834
NoOverflow (3 179 witnesses)	executed on		3 179	3 179
	uniquely confirmed		44	74
	jointly confirmed		870	870
MemSafety (4 426 witnesses)	executed on		4 426	4 426
	uniquely confirmed		398	173
	jointly confirmed		811	811

**Validator Configuration.** Since our architecture (cf. Fig. 1) allows for a wide range of verifiers to be used for validation, there are many interesting configurations for constructing a validator. Exploring all of these in order to find the best configuration, however, would require significant computational resources, and also be susceptible to over-fitting. Instead, we chose a heuristic based on the results of the competition from the previous year, i.e., SV-COMP 2019 [3]. The idea is that a verifier which performed well at *verifying* tasks for a specific specification is also a promising candidate to be used in *validating* results for that specification. Therefore the configuration of our validator  uses  as verifier for tasks with specification ReachSafety,  for NoOverflow and Termination, and  for MemSafety.

### Results

The results of the validation phase in SV-COMP 2020 [5] are summarized in Table 1 (for violation witnesses) and Table 2 (for correctness witnesses). For each specification,  was able to not only confirm a large number of results that were also validated by other tools, but also to confirm results that were not previously validated by any of the other tools.^6^

For violation witnesses, we can observe that  confirms significantly less witnesses than the other validators. This can be explained partially by the restrictive time limit of 90 s. Our approach not only adds overhead when generating the program from the witness, but this new program can also be harder to parse and analyze for the verifier we use in the backend. It is also the case that the verifiers that we use in  are not tuned for such a short time limit, as a verifier in the competition will always get the full 15 min. For specification ReachSafety, for example, we use  , which starts with a very simply analysis and switches verification strategies after a fixed time that happens to be also 90 s. So in this case we will never benefit from the more sophisticated strategies that  offers.

For validation of correctness witnesses, where the time limit is higher, this effect is less noticeable such that the number of results confirmed by  is more in line with the numbers achieved by the other validators. For specification MemSafety,  even confirms more correctness witnesses than  . This indicates that  was a good choice in our configuration for that specification.  generally performs much better in verification of MemSafety tasks than  , so this result was expected.

Before the introduction of  , there was only one validator for correctness witnesses in the categories NoOverflow and MemSafety, while constructing a validator for those categories with our approach did not require any additional development effort.

## Related Work

*Programs from Proofs.* Our approach for generating programs can be seen as a variant of the Programs from Proofs (PfP) framework [27, 41]. Both generate programs from an abstract reachability graph of the original program. The difference is that PfP tries to remove all specification violations from the graph, while we just encode them into the generated program as violation of the standard reachability property. We do this for the original specification and the invariants in the witness, which we treat as additional specifications.

*Automata-Based Software Model Checking.* Our approach is also similar to that of the validator  [10]. For violation witnesses, it also constructs the product of CFA and witness. For correctness witnesses, it instruments the invariants directly into the CFA of the program (see [10], Sect. 4.2) and passes the result to its verification engine, while  constructs the product of CFA and witness, and applies a similar instrumentation. In both cases,  ’s transformer produces a C program, which can be passed to an independent verifier.

*Reducer-Based Conditional Model Checking.* The concept of generating programs from an ARG has also been used to successfully construct conditional verifiers [14]. Our approach for correctness witnesses can be seen as a special case of this technique, where  acts as initial verifier that does not try to reduce the search space and instead just instruments the invariants from the correctness witness as additional specification into the program.

*Verification Artifacts and Interfacing.* The problem that verification results are not treated well enough by the developers of verification tools is known [1] and there are also other works that address the same problem, for example, the work on execution reports [19] or on cooperative verification [17].

*Test-Case Generation.* The idea to generate test cases from verification counterexamples is more than ten years old [8, 39], has since been used to create debuggable executables [31, 33], and was extended and combined to various successful automatic test-case generation approaches [24, 25, 29, 35].

*Execution.* Other approaches [18, 22, 28] focus on creating tests from concrete and tool-specific counterexamples. In contrast, witness validation does not require full counterexamples, but works on more flexible, possibly abstract, violation witnesses from a wide range of verification tools.

*Debugging and Visualization.* Besides executing a test, it is important to understand the cause of the error path [23], and there are tools and methods to debug and visualize program paths [2, 9, 26].

## Conclusion

We address the problem of constructing a tool for witness validation in a systematic and generic way: We developed the concept of *validation via verification*, which is a two-step approach that first applies a program transformation and then applies an off-the-shelf verification tool, without development effort.

The concept is implemented in the witness validator  , which has already been successfully used in SV-COMP 2020. The validation results are impressive: the new validator enriches the competition’s validation capabilities by 164 uniquely confirmed violation results and 834 uniquely confirmed correctness results, based on the witnesses provided by the verifiers. This paper does not contain an own evaluation, but refers to results from the recent competition in the field.

The major benefit of our concept is that it is now possible to configure a spectrum of validators with different strengths, based on different verification engines. The ‘time to market’ of new verification technology into validators is negligibly small because there is no development effort necessary to construct new validators from new verifiers. A potential technology bias is also reduced.

**Data Availability Statement.** All data from SV-COMP 2020 are publicly available: witnesses [7], verification and validation results as well as log files [5], and benchmark programs and specifications [6]^7^. The validation statistics in Tables 1 and 2 are available in the archive [5] and on the SV-COMP website^8^.  1.0 is available on GitLab^9^ and in our AEC-approved virtual machine [16].

## Footnotes

1 Latest version of standardized witness format: https://github.com/sosy-lab/sv-witnesses

2 https://sv-comp.sosy-lab.org/2020/systems.php

3 https://gitlab.com/sosy-lab/sv-comp/archives-2020/tree/master/2020

4 These invariants are the central piece of information in correctness witnesses. While invariants that proof a program correct can be hard to come up with, they are usually easier to check.

5 https://github.com/sosy-lab/sv-benchmarks/tree/svcomp20

6 In the statistics, a witness is only counted as confirmed if the verifier correctly stated whether the input program satisfies the respective specification.

7 https://github.com/sosy-lab/sv-benchmarks/tree/svcomp20

8 https://sv-comp.sosy-lab.org/2020/results/results-verified/validatorStatistics.html

9 https://gitlab.com/sosy-lab/software/metaval/-/tree/1.0

## References

[1] Alglave, J., Donaldson, A.F., Kröning, D., Tautschnig, M.: Making software verification tools really work. In: Proc. ATVA, LNCS, vol. 6996, pp. 28–42. Springer, Heidelberg (2011). 10.1007/978-3-642-24372-1_3

[2] Artho, C., Havelund, K., Honiden, S.: Visualization of concurrent program executions. In: Proc. COMPSAC, pp. 541–546. IEEE (2007). 10.1109/COMPSAC.2007.236

[3] Beyer, D.: Automatic verification of C and Java programs: SV-COMP 2019. In: Proc. TACAS (3), LNCS, vol. 11429, pp. 133–155. Springer, Cham (2019). 10.1007/978-3-030-17502-3_9

[4] Beyer, D.: Advances in automatic software verification: SV-COMP 2020. In: Proc. TACAS (2), LNCS, vol. 12079, pp. 347–367. Springer, Cham (2020). 10.1007/978-3-030-45237-7_21

[5] Beyer, D.: Results of the 9th International Competition on Software Verification (SV-COMP 2020). Zenodo (2020). 10.5281/zenodo.3630205

[6] Beyer, D.: SV-Benchmarks: Benchmark set of 9th Intl. Competition on Software Verification (SV-COMP 2020). Zenodo (2020). 10.5281/zenodo.3633334

[7] Beyer, D.: Verification witnesses from SV-COMP 2020 verification tools. Zenodo (2020). 10.5281/zenodo.3630188

[8] Beyer, D., Chlipala, A.J., Henzinger, T.A., Jhala, R., Majumdar, R.: Generating tests from counterexamples. In: Proc. ICSE, pp. 326–335. IEEE (2004). 10.1109/ICSE.2004.1317455

[9] Beyer, D., Dangl, M.: Verification-aided debugging: An interactive web-service for exploring error witnesses. In: Proc. CAV (2), LNCS, vol. 9780, pp. 502–509. Springer, Cham (2016). 10.1007/978-3-319-41540-6_28

[10] Beyer, D., Dangl, M., Dietsch, D., Heizmann, M.: Correctness witnesses: Exchanging verification results between verifiers. In: Proc. FSE, pp. 326–337. ACM (2016). 10.1145/2950290.2950351

[11] Beyer, D., Dangl, M., Dietsch, D., Heizmann, M., Stahlbauer, A.: Witness validation and stepwise testification across software verifiers. In: Proc. FSE, pp. 721–733. ACM (2015). 10.1145/2786805.2786867

[12] Beyer, D., Dangl, M., Lemberger, T., Tautschnig, M.: Tests from witnesses: Execution-based validation of verification results. In: Proc. TAP, LNCS, vol. 10889, pp. 3–23. Springer, Cham (2018). 10.1007/978-3-319-92994-1_1

[13] Beyer, D., Gulwani, S., Schmidt, D.: Combining model checking and data-flow analysis. In: Handbook of Model Checking, pp. 493–540. Springer, Cham (2018). 10.1007/978-3-319-10575-8_16

[14] Beyer, D., Jakobs, M.C., Lemberger, T., Wehrheim, H.: Reducer-based construction of conditional verifiers. In: Proc. ICSE, pp. 1182–1193. ACM (2018). 10.1145/3180155.3180259

[15] Beyer D, Löwe S, Wendler P (2017). Reliable benchmarking: Requirements and solutions. Int. J. Softw. Tools Technol. Transfer.

[16] Beyer, D., Spiessl, M.: Replication package (virtual machine) for article ‘MetaVal: Witness validation via verification’ in Proc. CAV 2020. Zenodo (2020). 10.5281/zenodo.3831417

[17] Beyer, D., Wehrheim, H.: Verification artifacts in cooperative verification: Survey and unifying component framework. arXiv/CoRR **1905**(08505), May 2019. https://arxiv.org/abs/1905.08505

[18] Cadar, C., Ganesh, V., Pawlowski, P.M., Dill, D.L., Engler, D.R.: EXE: Automatically generating inputs of death. In: Proc. CCS, pp. 322–335. ACM (2006). 10.1145/1180405.1180445

[19] Castaño, R., Braberman, V.A., Garbervetsky, D., Uchitel, S.: Model checker execution reports. In: Proc. ASE, pp. 200–205. IEEE (2017). 10.1109/ASE.2017.8115633

[20] Christakis, M., Bird, C.: What developers want and need from program analysis: An empirical study. In: Proc. ASE, pp. 332–343. ACM (2016). 10.1145/2970276.2970347

[21] Clarke, E.M., Grumberg, O., McMillan, K.L., Zhao, X.: Efficient generation of counterexamples and witnesses in symbolic model checking. In: Proc. DAC, pp. 427–432. ACM (1995). 10.1145/217474.217565

[22] Csallner, C., Smaragdakis, Y.: Check ‘n’ crash: Combining static checking and testing. In: Proc. ICSE, pp. 422–431. ACM (2005). 10.1145/1062455.1062533

[23] Ermis, E., Schäf, M., Wies, T.: Error invariants. In: Proc. FM, LNCS, vol. 7436, pp. 187–201. Springer, Heidelberg (2012). 10.1007/978-3-642-32759-9_17

[24] Godefroid, P., Klarlund, N., Sen, K.: Dart: Directed automated random testing. In: Proc. PLDI, pp. 213–223. ACM (2005). 10.1145/1065010.1065036

[25] Gulavani, B.S., Henzinger, T.A., Kannan, Y., Nori, A.V., Rajamani, S.K.: Synergy: A new algorithm for property checking. In: Proc. FSE, pp. 117–127. ACM (2006). 10.1145/1181775.1181790

[26] Gunter, E.L., Peled, D.A.: Path exploration tool. In: Proc. TACAS, LNCS, vol. 1579, pp. 405–419. Springer, Heidelberg (1999). 10.1007/3-540-49059-0_28

[27] Jakobs, M.C., Wehrheim, H.: Programs from proofs: A framework for the safe execution of untrusted software. ACM Trans. Program. Lang. Syst. **39**(2), 7:1–7:56 (2017). 10.1145/3014427

[28] Li, K., Reichenbach, C., Csallner, C., Smaragdakis, Y.: Residual investigation: Predictive and precise bug detection. In: Proc. ISSTA, pp. 298–308. ACM (2012). 10.1145/2338965.2336789

[29] Majumdar, R., Sen, K.: Hybrid concolic testing. In: Proc. ICSE, pp. 416–426. IEEE (2007). 10.1109/ICSE.2007.41

[30] McConnell RM, Mehlhorn K, Näher S, Schweitzer P (2011). Certifying algorithms. Comput. Sci. Rev..

[31] Müller, P., Ruskiewicz, J.N.: Using debuggers to understand failed verification attempts. In: Proc. FM, LNCS, vol. 6664, pp. 73–87. Springer, Heidelberg (2011). 10.1007/978-3-642-21437-0_8

[32] Plasil F, Visnovsky S (2002). Behavior protocols for software components. IEEE Trans. Software Eng..

[33] Rocha, H., Barreto, R.S., Cordeiro, L.C., Neto, A.D.: Understanding programming bugs in ANSI-C software using bounded model checking counter-examples. In: Proc. IFM, LNCS, vol. 7321, pp. 128–142. Springer, Heidelberg (2012). 10.1007/978-3-642-30729-4_10

[34] Schneider FB (2000). Enforceable security policies. ACM Trans. Inf. Syst. Secur..

[35] Sen, K., Marinov, D., Agha, G.: Cute: A concolic unit testing engine for C. In: Proc. FSE, pp. 263–272. ACM (2005). 10.1145/1081706.1081750

[36] Šerý, O.: Enhanced property specification and verification in Blast. In: Proc. FASE, LNCS, vol. 5503, pp. 456–469. Springer, Heidelberg (2009). 10.1007/978-3-642-00593-0_32

[37] Svejda, J., Berger, P., Katoen, J.P.: Interpretation-based violation witness validation for C: NitWit. In: Proc. TACAS, LNCS, vol. 12078, pp. 40–57. Springer, Cham (2020). 10.1007/978-3-030-45190-5_3

[38] Turing, A.: Checking a large routine. In: Report on a Conference on High Speed Automatic Calculating Machines, pp. 67–69. Cambridge Univ. Math. Lab. (1949)

[39] Visser, W., Păsăreanu, C.S., Khurshid, S.: Test-input generation with Java PathFinder. In: Proc. ISSTA, pp. 97–107. ACM (2004). 10.1145/1007512.1007526

[40] Wimmer, S., von Mutius, J.: Verified certification of reachability checking for timed automata. In: Proc. TACAS, LNCS, vol. 12078, pp. 425–443. Springer, Cham (2020). 10.1007/978-3-030-45190-5_24

[41] Wonisch, D., Schremmer, A., Wehrheim, H.: Programs from proofs: A PCC alternative. In: Proc. CAV, LNCS, vol. 8044, pp. 912–927. Springer, Heidelberg (2013). 10.1007/978-3-642-39799-8_65

